# The German S3 guideline on titanium hypersensitivity in implant dentistry: consensus statements and recommendations

**DOI:** 10.1186/s40729-022-00451-1

**Published:** 2022-11-04

**Authors:** Lena Katharina Müller-Heupt, Eik Schiegnitz, Sebahat Kaya, Elisabeth Jacobi-Gresser, Peer Wolfgang Kämmerer, Bilal Al-Nawas

**Affiliations:** 1 Department of Oral- and Maxillofacial Surgery, Plastic Surgery, University Medical Centre of the Johannes Gutenberg-University Mainz, Augustusplatz 2, 55131 Mainz, Germany; 2 Oral Surgery, Private Practice, Heidesheimer Straße 20, 55124 Mainz, Germany

**Keywords:** Allergy, Dental implant, Ceramic implant, Epicutaneous test, German guidelines, Hypersensitivity, Titanium implant intolerance, LTT, MELISA, Patch test, Pro-inflammatory cytokines, Superstructures, Titanium, Titanium dioxide

## Abstract

**Background:**

There is currently a lack of guidelines for clinicians regarding titanium hypersensitivity in implant dentistry. Diagnostic tests such as the epicutaneous test or the lymphocyte transformation test showed inconsistent results regarding reliability and validity and thus, evidence-based consensus recommendations regarding diagnostic and therapeutic options may be helpful in clinical decision-making. Therefore, the German S3 guideline on titanium hypersensitivity in implant dentistry was developed.

**Findings:**

In the objectives, procedure, voting method and venue were defined and the consensus participants were invited. A systematic literature research was performed, and the overall quality of the evidence was rated according to the GRADE working group. Eight recommendations were formulated within the framework of a structured consensus conference under independent moderation and could be voted on with strong consensus (> 95% agreement). The formulated statements and recommendations were developed in small groups according to the guidelines of the Association of the Scientific Medical Societies in Germany (AWMF) and were discussed and agreed upon in the plenum.

**Conclusions:**

For reasonable decision-making, a patient’s clinical symptoms should be regarded as leading parameters, which are usually expressed by a local inflammatory reaction with subsequent disturbed osseous integration. Allergy tests, such as the epicutaneous test or the lymphocyte transformation test are not helpful in titanium intolerance assessments, since these tests indicate T cell-mediated allergies, which are not observed in titanium intolerance reactions. Other metals and impurities that might be present in superstructures or alloys also need to be considered as the cause of an intolerance reaction and a trigger for contact sensitization. In the case of a suspected titanium particle-related, local immunologically induced inflammatory reaction with subsequent impaired osseous integration, dental ceramic implants can be considered as a therapeutic option.

**Graphical Abstract:**

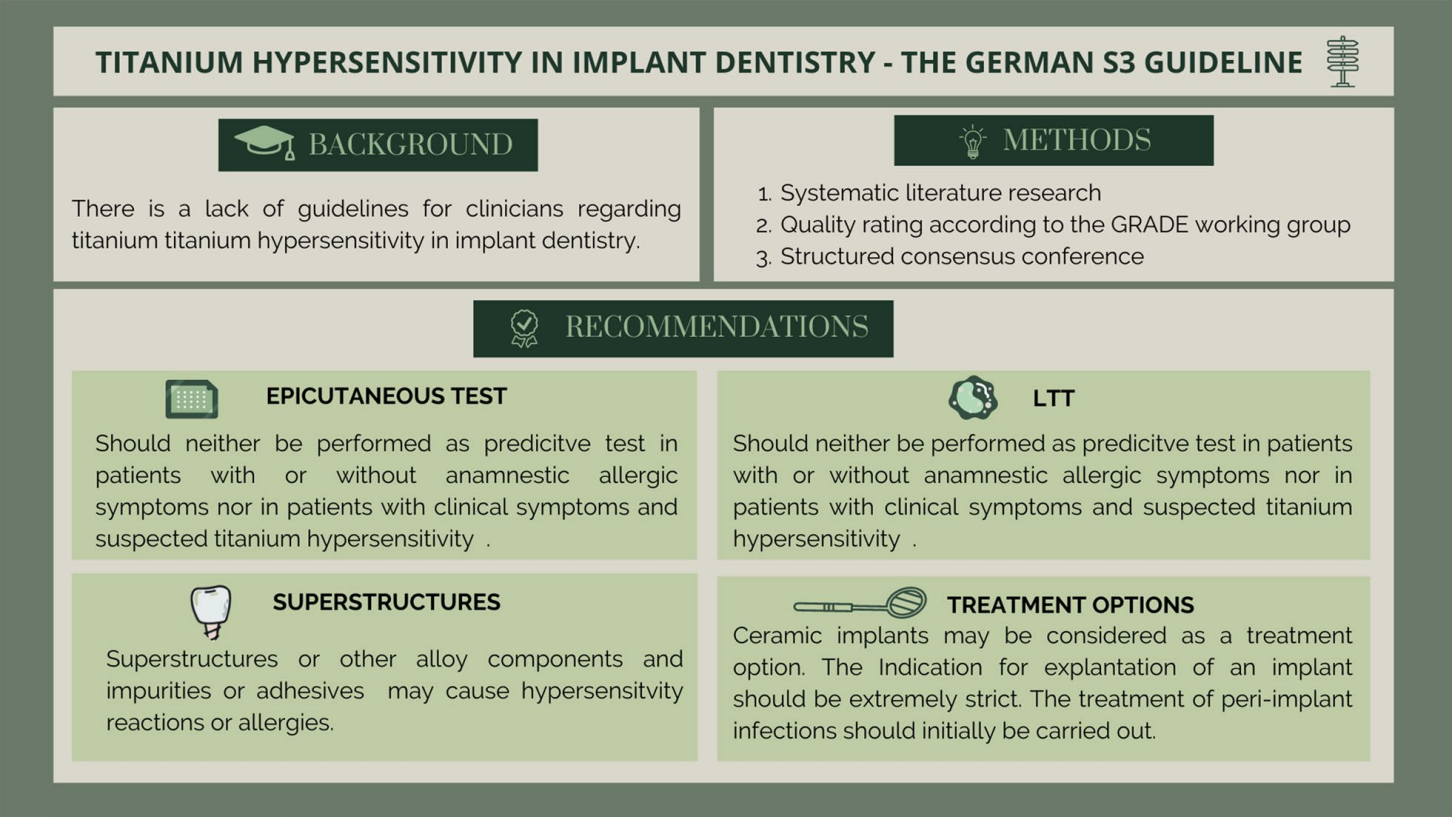

## Background

The demand for dental implants is continuously increasing due to the demographic change and an increasing esthetic and masticatory awareness within the population. However, with the increase in implantations, the number of cases with peri-implantitis is also increasing. With the development of the German S3 guideline on titanium hypersensitivity in implant dentistry, necessary recommendations regarding diagnostic and therapeutic options for patients with clinically suspicious intolerance reactions prior to and after implant insertion are provided as an evidence- and consensus-based decision support for dentists for the first time. The aim of this guideline was to determine in which cases patients would benefit from extended dermatological or laboratory-based diagnostics and how the clinical relevance of individual diagnostic findings and symptoms should be evaluated. The following PICO design was used: which effect does the insertion of a titanium dental implant (I) have in patients with and without metal allergy (P) compared to patients with ceramic implants or without implants (C) in terms of the development of a hypersensitivity reaction (O). A systematic literature search was performed, and the overall quality of the evidence was rated according to the GRADE working group. Randomized controlled trials were assessed according to the Cochrane Risk of Bias Tool I [1], cohort studies according to the Newcastle–Ottawa scale [2], and case series according to Moga et al. 2012 [3].

A total of eight recommendations were discussed and formulated in a structured consensus conference with an independent moderation. Strong consensus agreement was reached by > 95% of participants, consensus agreement by 75 to 95% of the participants, majority consensus agreement by 50 to 75% of participants and no consensus agreement by < 50% of the participants.

The formulated statements and recommendations were developed in small groups according to the guidelines of the Association of the Scientific Medical Societies in Germany (AWMF) and were afterwards discussed and agreed upon in the plenum.

A systematic review regarding diagnostic tests for titanium hypersensitivity was published by the authors and may be accessed for further background information regarding this topic [4].

## Consensus statements

### Predictive epicutaneous test (ECT) for titanium hypersensitivity

The ECT for the clarification of a potentially existing sensitization (referred to in the literature as prophetic testing) should not be performed (strong consensus/literature: [5, 6]/quality of evidence: moderate).

Background: The ECT, with which contact sensitization can be detected, plays a subordinate role for the question of titanium implant tolerance, due to its divergent pathophysiology compared to an allergy.

### Predictive ECT for titanium hypersensitivity in patients with anamnestic allergic symptoms

The ECT should also not be performed in patients with a history of relevant previous diseases (strong consensus/literature: [5–8]/quality of evidence: low).

The ECT, which detects contact sensitizations, plays a subordinate role for the question of titanium implant tolerance, due to differences in pathophysiology compared to an allergy.

### ECT in patients with clinical symptoms and suspected titanium hypersensitivity

The ECT should also not be performed in patients with suspected clinical intolerance (strong consensus/literature: [6, 7, 9]/quality of evidence: low).

The ECT, which can detect contact sensitization, plays a subordinate role for the question of titanium implant tolerance, since differences in the pathophysiology compared to an allergy are present.

### Predictive lymphocyte transformation test (LTT) with regard to titanium

The LTT for the clarification of a potentially existing sensitization to titanium (referred to in the literature as prophetic testing) should not be performed (strong consensus/literature [10]/quality of evidence: low).

The LTT, which can detect an allergic reaction in vitro, plays a subordinate role for the question of titanium implant tolerance, since this is not an allergy in the classical sense from a pathophysiological point of view.

### Predictive LTT regarding titanium in patients with anamnestic allergic symptoms

The LTT in relation to titanium should also not be performed in patients with a medical history of relevant previous diseases (strong consensus, literature: [10]/quality of evidence: low).

The LTT, which can detect an allergic reaction in vitro, plays a subordinate role for the question of titanium implant tolerance, due to differences in pathophysiology compared to an allergy.

### LTT in patients with clinical symptoms and suspected titanium hypersensitivity

LTT should also not be performed in patients with suspected clinical intolerance to titanium (strong consensus/literature [10]/quality of evidence: low).

The LTT, which can detect an allergic reaction in vitro, plays a subordinate role for the question of titanium implant tolerance, since differences in the pathophysiology compared to an allergy are present.

### Superstructures

Regarding hypersensitivity reactions in implant dentistry, it should be kept in mind that superstructures may cause hypersensitivity reactions or allergies. Other alloy components and impurities as well as adhesives should be considered as well (strong consensus/literature [11]/quality of evidence: low).

When discussing a metal allergy or titanium intolerance reaction, it must be kept in mind that endosseous implants are predominantly made of pure titanium (grade 4), but grade 5 titanium alloys and other alloys (metals) are also used, particularly in superstructures. An omission test can point the way to incompatibilities or allergies related to materials in superstructures. In the event of an allergic contact dermatitis of the oral mucosa to other prosthetic materials in implant alloys or superstructures (such as aluminum, vanadium, palladium or niobium, as well as nickel contamination) or other prosthetic materials in superstructures, such as cements or adhesives, LTT or ECT may be useful for differential diagnosis.

In vitro test methods should be considered, if epicutaneous testing is not possible for technical reasons (e.g., chronic generalized eczema) or if this would be too dangerous due to the toxicity of the substance, which should be taken into account for chemical substances (e.g., acrylates) that have been little investigated in this regard. In addition, the LTT has the advantage that an iatrogenic sensitization of the patient cannot take place, since the provocation with the allergen takes place in vitro and not within the patients skin [12].

### Treatment options for patients with suspected titanium hypersensitivity reaction

For patients with suspected titanium intolerance, dental ceramic implants may be considered as a treatment option (strong consensus/literature: [9, 13, 14]/quality of evidence: low).

The indication for an explantation of a titanium implant should only be determined very strictly. The treatment of peri-implant biofilm-associated infections should initially be carried out in accordance with national guidelines. Since no ceramic products are available for orthodontic anchorage screws, conventional anchorage tools should be used and tissue contact should be kept as short as possible.

To date, titanium intolerance has not been adequately documented in the literature and valid diagnostic evidence is questionable. However, studies show evidence that the inflamed environment (peri-implantitis/mucositis) is associated with a higher peri-implant titanium particle load. Macrophage stimulation tests attempt to analyze this individual immune response in vitro. It has also been shown that patients have genetic predispositions with regard to their individual inflammatory reaction, which can be detected in genetic tests. Currently available tests can therefore only be regarded as useful indicative diagnostic tools. The clinical differentiation between a triggering bacterial inflammation and possible immunological inflammation due to titanium particles is not yet distinguishable with strong evidence.

## Conclusion

For reasonable therapeutic decisions, clinical symptoms should be regarded as leading parameters, which are expressed by a local inflammatory reaction with subsequent disturbed osseous integration. Tests for the detection of type IV sensitizations, such as the ECT or the LTT, are not recommended since these tests indicate T cell-mediated allergies, which titanium intolerance does not represent. Furthermore, these tests are not target-specific in implant dentistry since titanium particle-induced peri-implant inflammation is a nonspecific immune activation. However, a specific immunological reaction in the sense of sensitization can be triggered by other metals used in titanium alloys, by impurities of the implant surface or in metal alloys of abutments and superstructures.

According to national guidelines, peri-implantitis treatment should be performed and the explantation of a dental implant should be regarded as ultimate option. Dental ceramic implants can be considered as a therapeutic option.

## Data Availability

The datasets used and/or analyzed during the current study are available from the corresponding author on reasonable request.
